# Exercise, Appetite and Weight Control: Are There Differences between Men and Women?

**DOI:** 10.3390/nu8090583

**Published:** 2016-09-21

**Authors:** Alice E. Thackray, Kevin Deighton, James A. King, David J. Stensel

**Affiliations:** 1 School of Sport, Exercise and Health Sciences, Loughborough University, Leicestershire LE11 3TU, UK; A.E.Thackray@lboro.ac.uk (A.E.T.); J.A.King@lboro.ac.uk (J.A.K.); 2 Institute for Sport, Physical Activity and Leisure, Leeds Beckett University, Leeds LS6 3QS, UK; K.Deighton@leedsbeckett.ac.uk

**Keywords:** appetite, appetite-regulatory hormones, compensation, energy balance, energy intake, exercise, sex-based differences, weight control

## Abstract

Recent years have witnessed significant research interest surrounding the interaction among exercise, appetite and energy balance, which has important implications for health. The majority of exercise and appetite regulation studies have been conducted in males. Consequently, opportunities to examine sex-based differences have been limited, but represent an interesting avenue of inquiry considering postulations that men experience greater weight loss after exercise interventions than women. This article reviews the scientific literature relating to the acute and chronic effects of exercise on appetite control in men and women. The consensus of evidence demonstrates that appetite, appetite-regulatory hormone and energy intake responses to acute exercise do not differ between the sexes, and there is little evidence indicating compensatory changes occur after acute exercise in either sex. Limited evidence suggests women respond to the initiation of exercise training with more robust compensatory alterations in appetite-regulatory hormones than men, but whether this translates to long-term differences is unknown. Current exercise training investigations do not support sex-based differences in appetite or objectively assessed energy intake, and increasing exercise energy expenditure elicits at most a partial energy intake compensation in both sexes. Future well-controlled acute and chronic exercise studies directly comparing men and women are required to expand this evidence base.

## 1. Introduction

Obesity is a major risk factor for several chronic diseases, including type 2 diabetes mellitus and cardiovascular disease, and remains a significant global burden from a public health and economic standpoint [1,2]. Weight loss as little as 3% of initial body mass is sufficient to promote favourable changes in several chronic disease risk markers and can be accomplished by increasing energy expenditure through exercise and/or reducing energy intake to achieve a sustained negative energy balance [3]. Recent years have witnessed significant research interest surrounding the interaction between exercise, appetite and energy balance, which has direct implications for the implementation of exercise as a weight management strategy [4].

Similar to many scientific fields, the majority of exercise and appetite regulation studies have traditionally focused research efforts on men. Consequently, much less is known about the interaction between exercise and appetite in women, and the opportunity to examine potential sex-based differences has been limited. A handful of exercise training studies have demonstrated that men experience greater reductions in body mass and body fat than women [5,6,7], although this is not a universal finding [8,9]. Authors supporting the concept of divergent weight loss outcomes have suggested that women demonstrate greater compensatory responses to exercise by more accurately balancing energy intake and expenditure in order to defend body fat stores and preserve reproductive function [10,11,12].

Exercise-induced changes in hormones implicated in appetite control and energy balance (e.g., acylated ghrelin, peptide YY (PYY), glucagon-like peptide-1 (GLP-1), insulin, and leptin) may contribute to sex-based differences in body fat loss after exercise [13]. Although based on a limited number of studies, a previous review concluded that women exhibit compensatory changes in appetite ratings and hormones conducive to appetite stimulation; a response that is not seen in men [11]. However, this conclusion has not been supported by more recent experimental studies, which have documented similar appetite, appetite-regulatory hormone and energy intake responses to acute and chronic exercise-induced energy deficits in men and women [8,14,15].

The purpose of this article is to review recent developments regarding appetite, appetite-regulatory hormone and energy intake responses to single bouts of exercise (acute responses) and exercise training (chronic responses) in men and women. Furthermore, this review will consider the potential implications of these findings for health and highlight important areas for future research.

## 2. Appetite-Regulatory Hormones

Appetite and energy intake are regulated at the physiological level by the neuroendocrine system, which involves complex interactions between central and peripheral mediated pathways [16,17]. Appetite-regulatory hormones include episodic gut signals that are sensitive to short-term fluctuations in feeding behaviour and control hunger and satiety on a meal-to-meal basis (e.g., acylated ghrelin, PYY, and GLP-1), and tonic hormonal signals that regulate long-term changes in energy balance and body fat (e.g., insulin, and leptin). A brief introduction to these hormones is presented here, but the interested reader is directed to a number of comprehensive reviews documenting the precise role of these hormones in the homeostatic regulation of appetite and energy balance [16,17,18,19].

Of the short-acting appetite regulatory signals, ghrelin is unique as the only known gut peptide that is orexigenic, and is predominantly secreted into the circulation by the oxyntic glands of the stomach. Ghrelin exists in the circulation in two forms (acylated and unacylated) and, although only 10%–20% of circulating ghrelin is acylated ghrelin, this form is believed to be solely responsible for appetite stimulation [20]. Circulating ghrelin concentrations increase preprandially and are rapidly suppressed postprandially on a meal-to-meal basis. This temporal pattern of fluctuation is indicative of an important role in coordinating meal initiation [21].

Working in opposition to ghrelin, on a meal-to-meal basis, several appetite-inhibiting hormones serve to promote post-meal satiation and satiety (e.g., PYY, GLP-1, cholecystokinin, pancreatic polypeptide, and amylin). Of primary relevance to this review, PYY is predominantly synthesised and secreted from the intestinal L-cells and is present peripherally in two forms (PYY_1–36_ and PYY_3–36_), with PYY_3–36_ representing the most abundant and biologically active form. Concentrations of PYY are low in the fasted state and increase rapidly after meal intake, which highlights a potential role in meal termination and sensations of fullness between meals. Glucagon-like peptide-1 is also secreted from the intestinal L-cells in response to nutrient intake and similarly contributes to meal termination and satiety. It exists as an active (GLP_7–36_) and inactive (GLP_9–37_) form, with the active form rapidly degraded to its inactive form upon secretion into the circulation. The appetite-inhibiting effect of these hormones is further supported by studies demonstrating that peripheral administration of PYY_3–36_ [22] and GLP-1 [23] stimulates satiety and reduces ad libitum food intake in lean and obese individuals.

Leptin, secreted primarily from adipocytes, and insulin, released by the beta cells of the pancreas, are important regulators of energy balance, which are implicated in the long-term control of food intake and energy expenditure. Leptin and insulin are secreted in concentrations proportional to body fat mass, and act directly on the hypothalamus and other brain regions to exert anorexigenic effects. Circulating leptin and insulin concentrations are elevated in obese individuals, suggesting that a degree of resistance to the anorexigenic effects of these hormones may occur with obesity. This is further supported by evidence that the accumulation of adipose tissue weakens the inhibitory effect of fat mass on energy intake [24,25].

## 3. Exercise and Weight Loss

Exercise is an important component of weight management [3], and promotes a myriad of health benefits independent of weight loss [26]. It is well documented that exercise typically results in modest weight loss that can be enhanced when exercise is combined with dietary modifications [27,28]. However, the efficacy of exercise as a successful strategy for weight management varies markedly between individuals [29]. Interestingly, it has been suggested that sex may be a primary factor that affects the ability of structured exercise to promote weight loss and/or facilitate weight management [30].

The strongest evidence of a sex-based difference in the weight loss response to exercise was provided in the Midwest Exercise Trial by Donnelly and colleagues [6]. This study involved a 16-month supervised exercise training program at a set intensity and duration (five days per week, 20–45 min per session at 55%–70% peak oxygen uptake ($\dot{V}O$_2peak_)) with ad libitum diet in previously sedentary men and women. After the exercise intervention, men lost an average of 5.2 kg in body weight and 4.9 kg in fat mass, whereas women maintained body weight and fat mass. Other studies have also demonstrated that men experience greater weight loss than women in response to a supervised program of exercise when exercise is prescribed at a similar duration and relative exercise intensity across the sexes [5,31,32].

However, in many of these studies, the exercise-induced energy expenditure was substantially greater in men than women. This has been suggested as a potential reason for the reported sex-based differences in exercise-induced weight loss [33], in accordance with evidence that the energy expenditure of exercise is the strongest predictor of fat loss during an exercise program [34,35]. Exercise training studies prescribing exercise based on energy expenditure have reported comparable body composition changes in response to the training stimulus in men and women [8,9,36]. Specifically, Donnelly and colleagues [9] have published findings from a subsequent randomised controlled trial as a follow-up to the Midwest Exercise Trial in which the exercise-induced energy expenditure was matched between men and women over a 10-month supervised aerobic exercise training intervention. In contrast to their earlier study [6], when the energy expenditure was equivalent between the sexes, similar reductions in body weight and body fat were seen between men and women [9].

A common finding in the literature is the degree of individual variation in the weight loss response to exercise training in both sexes [8,9,29,35,37,38]. It has been suggested that individual differences in compensatory behaviours that negate the exercise-induced energy deficit may be responsible for this variability [29]. Specifically, evidence of increased hunger and energy intake have been reported in individuals who experience a lower than expected weight loss after a period of exercise training [29,37,38]. Consequently, studies investigating the effect of exercise on appetite regulation (appetite perceptions, appetite-regulatory hormones, energy intake) in men and women are important and will be discussed in the following sections of this review.

## 4. Acute Effects of Exercise on Appetite, Appetite-Regulatory Hormones and Energy Intake

A plethora of studies have been conducted examining the appetite, appetite-regulatory hormone and energy intake responses to acute exercise in men, and to a much lesser extent, women. This research has been reviewed in detail elsewhere [4,39,40,41,42,43], but a brief synopsis of the most pertinent studies is presented in this article to frame the research literature which has examined sex-based differences.

### 4.1. Appetite and Appetite-Regulatory Hormones

The consensus of evidence in healthy, normal weight men suggests that acylated ghrelin concentrations are transiently suppressed, and satiety hormones, most notably PYY and GLP-1, are elevated during and immediately after an acute bout of exercise. Such hormonal changes often coincide with a transient reduction in subjective appetite responses, which has been described as “exercise-induced anorexia” [44]. These responses become apparent when acute exercise is performed ≥60% of $\dot{V}O$_2peak_ typically [45,46,47,48,49], and have been replicated during a variety of exercise modes including running [45,46,48], cycling [47,50,51,52,53], swimming [54], resistance exercise [46,55] and high-intensity interval exercise [52,53,56]. Circulating appetite-regulatory hormones and appetite ratings typically return to control values within 30 to 60 min of exercise completion [39,46,48]; however, compensatory increases in appetite have been reported in some studies [52,54,57]. Furthermore, current evidence suggests that acute exercise elicits similar appetite and appetite-regulatory hormone responses in lean and overweight men [47], and does not stimulate compensatory changes in those who are overweight or obese [47,58].

Despite postulations that sex-based differences in appetite regulation may exist to enable women to preserve energy balance and reproductive function [10,11,12], several acute studies conducted in women suggest that they respond similarly to men. Specifically, transient alterations in appetite and appetite-regulatory hormone concentrations (acylated ghrelin, PYY_3–36_, and GLP-1) have been reported in a direction expected to suppress appetite in healthy, recreationally active [15], endurance-trained [59] and overweight and obese [60] women. Furthermore, the majority of studies report no evidence of compensatory increases in appetite perceptions and appetite-regulatory hormones up to 7.5 h after a single bout of exercise in women [15,59,60,61,62].

However, exceptions have been observed in the literature with some studies demonstrating that women do not exhibit an acute exercise-induced suppression of appetite [62,63,64] or changes in appetite-regulatory hormones [61,62]. Furthermore, in contrast to the aforementioned studies in men and women, Larson-Meyer and colleagues [64] reported an increase in acylated ghrelin concentrations during the 2 h period after 60 min running at 70% $\dot{V}O$_2peak_. Such discrepancies are likely related to differences in the exercise intensity, training status of participants, completion of exercise in the fasted or postprandial state, timing of meal intake and analytical methods used to quantify hormone concentrations.

Sex-based differences in the regulation of appetite in response to acute exercise have been examined directly in four studies [14,15,65,66]. The first acute exercise and appetite study that compared men and women was published by Kawano and colleagues [65]. The authors reported that 20 min of rope skipping exercise increased ratings of subjective hunger 30 min after exercise in women but not men; however, the absence of a control condition in this study and the somewhat unusual mode of exercise make this finding difficult to interpret. Furthermore, this study did not control for the potential confounding effects of the menstrual cycle, which represents an important consideration for acute exercise studies comparing men and women. In this regard, recent evidence suggests that compared with untailored programs, synchronising diet and exercise training interventions around the hormonal changes that occur during the menstrual cycle elicits greater weight loss [67] and improvements in muscle strength [68]. In addition, cyclical fluctuations in sex hormones (estrogen and progesterone) have been shown to alter appetite-regulatory hormone concentrations and energy intake in women across the menstrual cycle [69,70]. However, whether appetite responses to exercise in women are influenced by the menstrual cycle phase is not known and represents a research avenue to consider in the future. 

Subsequent studies directly comparing men and women have also incorporated measures of appetite-regulatory hormones and energy intake (discussed below) alongside subjective appetite perceptions to provide a more comprehensive picture of potential sex-based differences in appetite regulation. In this regard, Hagobian and colleagues [14] examined the appetite and hormonal responses to a single bout of cycling performed at 70% $\dot{V}O$_2peak_ until 30% of total daily energy expenditure was expended in healthy men and women matched for age and cardiorespiratory fitness. Importantly, the female participants were all studied during the early follicular phase of the menstrual cycle. The authors reported that appetite perceptions and appetite-regulatory hormone concentrations (acylated ghrelin and PYY_3–36_) were not different during the 40 min after exercise in either sex. Similarly, in another acute study, breaking up prolonged sitting with light- or moderate-intensity walking did not alter appetite or concentrations of acylated ghrelin and total PYY over the 5 h observation period in either sex [66]. The walking interventions adopted in this study comprised a total of 28 min walking performed in 2 min bouts every 20 min. This intermittent pattern of exercise contrasts with the vast majority of acute exercise and appetite studies, which have reported transient perturbations in appetite and appetite-regulatory hormones in response to continuous, moderate- to high-intensity exercise protocols. Indeed, the authors recognise that the exercise stimulus may have been insufficient (in intensity and duration) to provoke transient changes in appetite and appetite-regulatory hormones.

**Figure 1:**
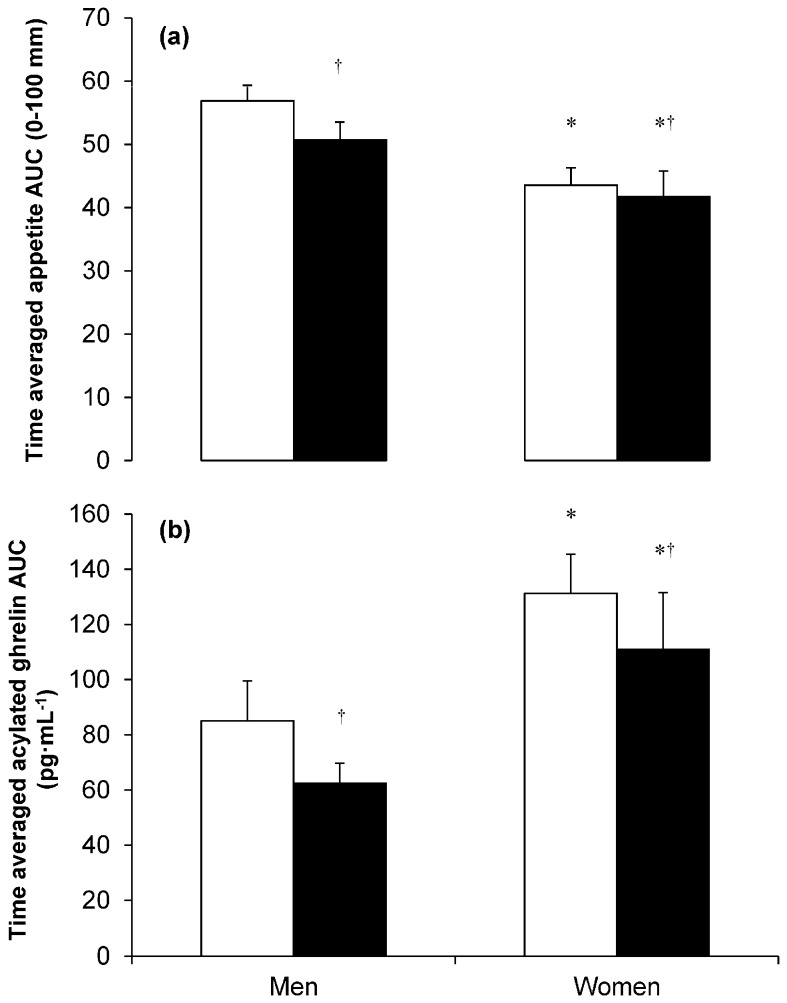
Time averaged total area under the curve (AUC) for appetite ratings (**a**); and plasma acylated ghrelin concentrations (**b**) in the control (□) and exercise (■) conditions. Each condition was 7 h and a single bout of exercise was performed between 0 to 1 h in the exercise condition (60 min running at 70% peak oxygen uptake). ^†^ Significant difference between exercise and control *p* ≤ 0.05; * Significant difference between women and men *p* ≤ 0.05. Values are mean (SEM), appetite ratings: *n* = 10 men, *n* = 10 women; acylated ghrelin: *n* = 8 men, *n* = 8 women. Data reproduced from reference [15]. © Wolters Kluwer Health, Inc. Reproduced with permission.

Recently, Alajmi and colleagues [15] examined the effect of 60 min treadmill running at 70% $\dot{V}O$_2peak_ on appetite and acylated ghrelin concentrations over 7 h in healthy men and women (studied during the follicular phase of the menstrual cycle). Despite the greater net energy expenditure during exercise in the men (3971 vs. 2536 kJ in men and women, respectively), both men and women exhibited an equivalent suppression in appetite and acylated ghrelin concentrations in response to acute exercise (Figure 1), with no evidence of compensatory responses to exercise in the 7 h observation period in either sex. Interestingly, the female participants in this study exhibited significantly greater acylated ghrelin concentrations compared with men. However, the relevance of this difference is unclear given subjective appetite ratings were greater in men than women. Furthermore, despite the greater appetite and lower acylated ghrelin concentrations in men than women, the appetite and acylated ghrelin responses to exercise were similar between the sexes.

### 4.2. Energy Intake

Many of the studies highlighted above included an ad libitum meal in the post-exercise period to assess potential changes in energy intake after a single exercise stimulus. The majority of studies in men report no change in absolute energy intake after acute exercise when a single or multiple ad libitum meals are provided 30 min to 7.5 h after the cessation of exercise [48,49,52,53,55,71,72,73]; however, some studies have reported increases [50,74] or decreases [47,58,75] in energy intake after acute exercise. Nevertheless, two studies have demonstrated that 24 h energy intake is unchanged after acute exercise in healthy men quantified from laboratory-based ad libitum meals and overnight food bags [48,52].

Similarly, evidence suggests that ad libitum energy intake remains unchanged in response to acute exercise in healthy women [64,76,77,78] and overweight and obese women [61,62,76]. As an exception, Larson-Meyer and colleagues [64] reported that absolute energy intake (ad libitum meal provided 120 min after exercise) was unchanged after 60 min running at 70% $\dot{V}O$_2peak_, but was increased after 60 min walking performed at the same relative intensity in a different group of women. The strength of this evidence is limited however by the between-measures design and the stark differences in body composition and cardiorespiratory fitness between the two groups. In another study, Pomerleau and colleagues [79] reported that ad libitum energy intake was increased 1 h after brisk walking at 70% $\dot{V}O$_2peak_ in healthy, young women. However, this change did not translate to altered energy intake over the remainder of the day after the provision of an ad libitum meal 6.5 h after exercise and an overnight snack bag. This highlights the importance of monitoring feeding behaviour over longer time periods.

Regardless of whether absolute energy intake remained unchanged, increased or decreased in response to acute exercise in the studies cited thus far, relative energy intake (total energy intake minus net energy expenditure of exercise) is invariably lower after exercise compared with control in men and women. Whilst this suggests that the exercise-induced energy deficit is maintained after exercise, which may have significant implications for weight management, it should be noted that the short-term follow up in these studies prevents us from drawing conclusions about behavioural and physiological responses over a greater period of time.

Studies directly comparing men and women have demonstrated that total energy intake is greater in men compared with women [14,15], but this difference disappears after adjustment for lean body mass [15]. These findings coupled with the higher appetite ratings reported in men in the study conducted by Alajmi and colleagues [15] lend support to the theory that lean body mass, as the largest contributor to resting metabolic rate, is a primary determinant of appetite control and energy intake [24,25].

**Figure 2:**
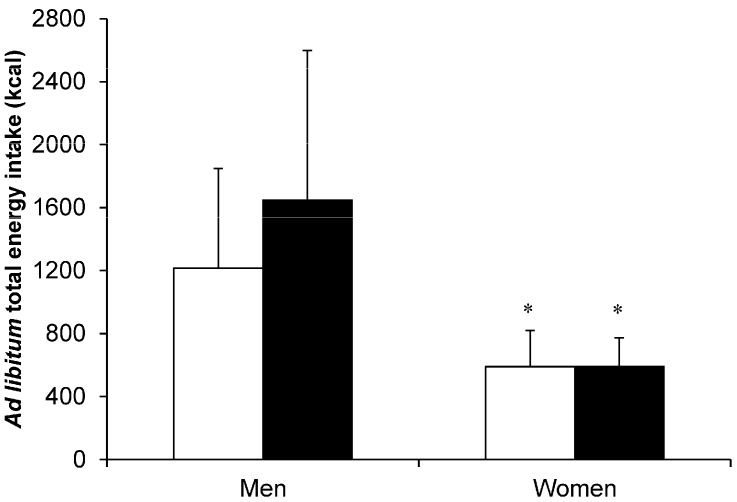
Total ad libitum energy intake during a single laboratory-based buffet meal in the control (□) and exercise (■) conditions in 11 men and 10 women. Exercise involved a single bout of cycling performed at 70% peak oxygen uptake until 30% of total daily energy expenditure was expended. * Significant difference between women and men *p* ≤ 0.05. Values are mean (SD). Data from reference [14]. © 2008 Canadian Science Publishing or its licensors. Reproduced with permission.

In addition to the appetite and hormone responses discussed in the previous section, Hagobian and colleagues [14] reported that absolute energy intake was unchanged in response to a single bout of cycling inducing a similar energy expenditure (30% of total daily energy expenditure) in men and women (energy expenditure: men, 975 kcal; women, 713 kcal) (Figure 2). The authors observed large variability in the energy intake responses (note large SDs on Figure 2 especially for men) with evidence of both higher and lower energy intake after exercise compared with a resting control condition in both men and women, which supports previous acute exercise and appetite regulation studies in healthy weight [78] and overweight and obese [62] women. Although the authors reported no significant change in energy intake after acute exercise in men or women, it is worth noting that mean ad libitum energy intake was higher in men after exercise (Figure 2) [14]. A closer examination of the mean differences and estimated standardised effect sizes revealed that energy intake after the exercise bout was 432 kcal higher than control in men (effect size = 0.68 indicating a moderate to large effect) compared with a 1 kcal increase after exercise in women (effect size = 0.004 indicating a trivial effect) (Figure 2). While this opposes the hypothesis that women are more likely to compensate for acute exercise-induced energy deficits by increasing energy intake, the conclusion that energy intake was unchanged in men should perhaps be interpreted with caution.

Subsequent studies investigating potential sex-based differences have reported no change in absolute energy intake in response to a single bout of running [15] and accumulating short bouts of walking to break up sedentary time [66]. Furthermore, these studies have consistently reported a lower relative energy intake after acute exercise compared with control in both sexes, suggesting that acute exercise suppressed relative energy intake independent of sex [14,15,66].

## 5. Chronic Effects of Exercise on Appetite, Appetite-Regulatory Hormones and Energy Intake

Although acute exercise studies provide important information regarding appetite regulation, exercise training studies are required to discern the long-term effects of exercise on energy balance and weight control. Exercise training studies are now reviewed with continued focus on appetite sensations, appetite-regulatory hormones and energy intake responses between men and women. It should be noted that few well-controlled exercise training studies have been conducted with many studies inherently limited by methodological constraints such as unsupervised exercise, self-reported energy intake, low exercise-induced energy expenditure and a lack of objective measures to quantify exercise energy expenditure.

### 5.1. Appetite and Appetite-Regulatory Hormones

Alterations in ghrelin concentrations after chronic exercise have been reported in conjunction with favourable changes in body weight. Specifically, weight loss in response to an exercise intervention has been shown to elevate total ghrelin concentrations in healthy weight and overweight and obese women in the fasted state and postprandially (reviewed by [40]). In contrast, Guelfi and colleagues [80] reported no effect of 12 weeks of aerobic or resistance exercise on fasting and postprandial hunger and concentrations of acylated ghrelin and PYY in overweight and obese men, despite a reduction in body fat mass in both exercise interventions. However, the authors reported lower fasting and postprandial leptin concentrations after exercise, which has been observed in other studies with men and women after exercise-induced weight loss [81,82]. Furthermore, chronic exercise studies resulting in weight loss in women appear to reduce fasting insulin concentrations [83,84], but have little effect on fasting total PYY and GLP-1_7–36_ concentrations [84,85].

Several exercise and appetite training studies recruiting both men and women have presented findings with the data for men and women combined [36,86,87]. Although these studies are informative, it is not possible to elucidate the direct effect of sex on the observed responses. Nevertheless, short-term exercise training without weight loss (1 h of daily walking at 70% $\dot{V}O$_2peak_ for 15 days) resulted in no changes in appetite or circulating concentrations of total PYY and insulin in obese men and women [87]. Martins and colleagues [36,86] have performed two studies investigating appetite and appetite-regulatory hormone responses to standardised meals in overweight and obese men and women undertaking 12 weeks of supervised aerobic exercise resulting in weight loss. The exercise intervention reduced fasting insulin concentrations but resulted in an increase in fasting acylated ghrelin concentrations and hunger perceptions [36]. In the postprandial state, circulating insulin was reduced along with a greater suppression in acylated ghrelin and a tendency for increased PYY and GLP-1 concentrations compared with levels before the intervention [36]. Furthermore, fasting and postprandial concentrations of leptin were reduced after the exercise intervention [86]. These findings led the authors to conclude that in response to chronic exercise, overweight individuals may balance the increased drive to eat with a concomitant increase in the satiety response to a meal, which supports previous findings in overweight and obese men [38].

In many of the studies discussed thus far, participants maintained their usual diet and subsequently lost body mass and fat mass by the end of the chronic exercise intervention. Therefore, it is difficult to determine whether the reported exercise-induced changes in appetite control are attributable to weight loss or to exercise training *per se*. In this regard, the study by Kanaley and colleagues [87] discussed previously did not report changes in appetite or appetite-regulatory hormones (total PYY, and insulin) in response to short-term exercise training without weight loss. Furthermore, total ghrelin concentrations were unchanged after exercise training in women who did not experience weight loss [85,88], and a study conducted in overweight adolescents observed no changes in fasting acylated ghrelin when body weight remained stable during the eight-month supervised exercise intervention [89]. Therefore, it is likely that alterations in appetite-regulatory hormones arise as a secondary consequence to changes in body mass.

**Figure 3:**
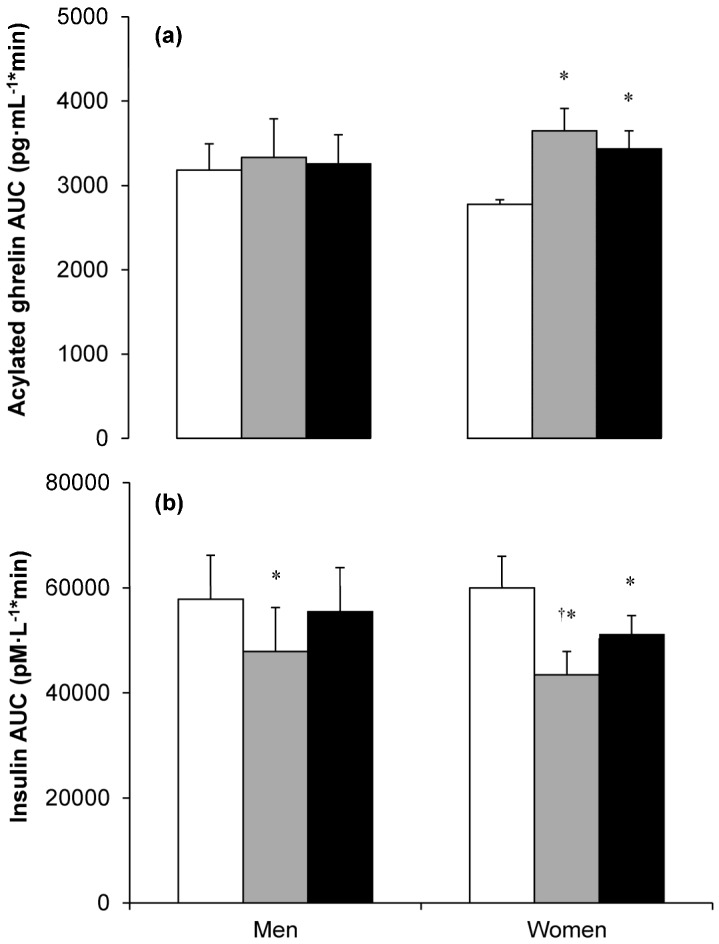
Total area under the curve (AUC) for plasma acylated ghrelin (**a**) and insulin (**b**) concentrations in the control (□), exercise with energy deficit (■) and exercise with energy balance (■) conditions in nine men and nine women. Exercise involved four consecutive days of treadmill exercise at 50%–65% peak oxygen uptake until 30% of total daily energy expenditure was expended. * Significant difference between exercise intervention and control; ^†^ Significant difference between exercise with energy deficit and exercise with energy balance. Values are mean (error bars not stated in original article). Data reproduced from reference [13]. © The American Physiological Society. Reproduced with permission.

Early evidence of exercise-induced sex differences in appetite hormones was provided by Hickey and colleagues [90]. In this study, 12 weeks of aerobic exercise training, without a change in body mass or body fat, significantly reduced fasting insulin and leptin concentrations in women but not in men. Subsequently, Hagobian and colleagues [13] examined appetite hormone responses to meal intake before and after four consecutive days of exercise in previously sedentary overweight and obese men and women. Daily aerobic exercise was performed on a treadmill at 50%–65% $\dot{V}O$_2peak_ resulting in an energy expenditure equivalent to ~30% of total daily energy expenditure and was completed with and without dietary replacement of the exercise-induced energy deficit. The authors reported that acylated ghrelin concentrations were higher and insulin concentrations were lower after both exercise interventions in women (Figure 3). In contrast, although men demonstrated lower insulin concentrations in the energy deficit condition, this effect was eliminated with energy replacement and acylated ghrelin was not different after exercise regardless of energy status (Figure 3). These findings suggest that women experience perturbations in appetite-regulatory hormones conducive to appetite stimulation in response to the initiation of exercise training. This is consistent with the hypothesis that the mechanisms governing energy balance are more tightly regulated in women than men.

However, in the Midwest Exercise Trial, lower insulin concentrations were observed in men but not women after the 16-month exercise training intervention [32]. This was accompanied by a divergent weight loss response to exercise training (discussed previously) which, coupled with the greater exercise energy expenditure in men, is likely to explain the differential insulin findings between this investigation and that of Hagobian and colleagues [13].

Although replacing the exercise-induced energy deficit suppressed appetite perceptions in men but not women, appetite was not altered when the energy deficit was maintained in either sex [13]. This supports a previous study reporting no change in postprandial appetite in response to 14 days of moderate- or high-intensity exercise training in lean men and women [91]. In another study, sex-based differences in body weight and appetite were examined in response to a 12-week supervised aerobic exercise intervention in overweight and obese men and women [8]. The 12-week exercise program resulted in similar reductions in body mass and body fat in the male and female participants. Furthermore, although fasting hunger ratings were elevated after the exercise training intervention, the magnitude of change was similar between the sexes and this difference did not translate to altered hunger responses in the postprandial period.

### 5.2. Energy Intake

Current evidence suggests that increasing energy expenditure during short-term exercise training (3 to 14 days) elicits partial compensations in energy intake [91,92,93,94]. Furthermore, a recent systematic review concluded that longer term exercise training studies (>2 weeks to 18 months) typically observe no change in energy intake across the training intervention [95]. However, the authors recognised that the available literature is prone to various methodological shortcomings as highlighted previously (e.g., unsupervised exercise, self-reported energy intake) which makes it difficult to interpret the findings with confidence.

A recent study directly comparing isoenergetic three-day energy deficits imposed by diet or exercise reported that dietary restriction stimulated a compensatory increase in ad libitum energy intake that was not observed in response to exercise [96]. This supports the findings from acute studies demonstrating rapid compensatory changes (appetite, appetite-regulatory hormones, energy intake) in response to diet-, but not exercise-induced energy deficits in men [71] and women [15]. These findings suggest that dietary restriction may represent a greater challenge to appetite regulation and energy balance than exercise, highlighting the importance of exercise to facilitate weight management in men and women [3].

A potential sex difference in energy intake responses during short-term exercise training was uncovered in two separate studies by Stubbs and colleagues [93,97]. Specifically, the authors reported that increasing energy expenditure through exercise training (seven days daily moderate- or high-intensity exercise) resulted in a partial compensation in energy intake in healthy women that equated to ~33% of the additional exercise-induced energy expenditure [93]. In contrast, there was no compensation in the energy intake response to an identical training stimulus in healthy men [97]. However, it is worth noting that energy intake was self-recorded in these studies through subjective dietary records and self-weighed intakes. This method of recording energy intake is particularly susceptible to participant bias, which makes it challenging to reconcile self-reported food intake with actual intake [98].

In a subsequent study adopting objective measures of both energy intake and energy expenditure, evidence of partial compensations in energy intake emerged after 14 days of supervised daily exercise which was equivalent to ~30% of the exercise-induced energy deficit [91]. This response was observed when energy intake data were combined for men and women, but only reached significance in men when analysed independently by sex [91]. This is consistent with an early study demonstrating that men, but not women, increase energy intake in response to five days of daily exercise, yet neither sex fully compensated for the imposed exercise energy expenditure [92]. Consequently, these findings refute the hypothesis that women compensate for chronic exercise-induced energy deficits by increasing energy intake.

When data is combined for men and women, studies investigating the effect of 12 weeks of supervised exercise (five days·week^−1^, 500 kcal·session^−1^) on body composition and appetite control have reported no exercise-induced change in energy intake assessed using self-reported food diaries [36] or laboratory-based test meals [99]. Furthermore, Westerterp and colleagues [5] reported no significant change in energy intake assessed using a self-reported seven-day weighed diary in men or women after 40 weeks of endurance training. In the Midwest Exercise Trials, energy intake was assessed using a combination of ad libitum meals in the University cafeteria and 24 h recall in overweight and obese men and women undergoing a supervised aerobic exercise program for 10 [9] or 16 months [6]. Similarly, no difference in energy intake was reported after the exercise training interventions in either sex [6,9].

**Figure 4:**
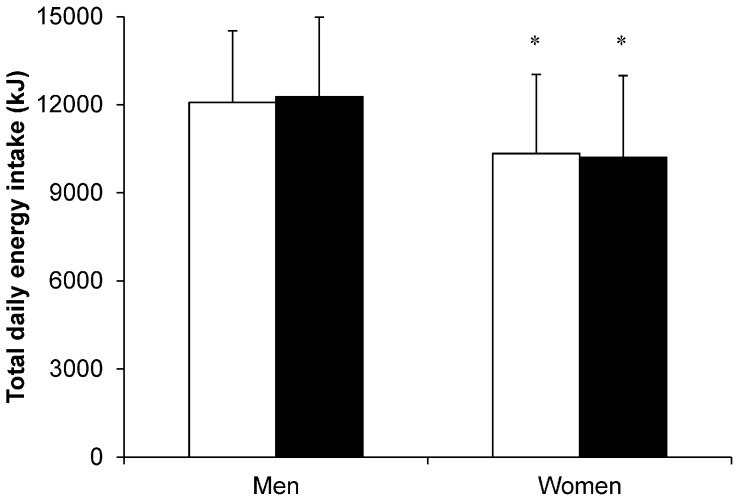
Total daily energy intake before (□) and after (■) a 12-week aerobic exercise training intervention in overweight and obese men (*n* = 35) and women (*n* = 72). Total daily energy intake was quantified objectively using laboratory-based test meal days at Weeks 0 and 12. On each day, participants were provided with an individualised fixed-energy breakfast (ad libitum at Week 0), fixed-energy lunch, ad libitum dinner and evening snack box. * Significant difference between women and men *p* ≤ 0.05. Values are mean (SD). Data from reference [8]. © Wolters Kluwer Health, Inc. Reproduced with permission.

When exercise is supervised and energy intake is quantified objectively using laboratory-based ad libitum meals, no changes in daily energy intake were observed in overweight and obese men or women after a 12-week aerobic exercise intervention (Figure 4) [8]. The authors of this study also highlighted the large variability in individual weight loss responses, both in magnitude and direction, which may afford some insight into why many individuals do not achieve their predicted changes in body composition with chronic exercise. Such heterogeneity in response to alterations in energy balance has been recognised previously [8,9,29,35,37,38]. Interestingly and pertinent to this review, overweight and obese men and women typically demonstrate a similar degree of individual variability when the exercise-induced energy expenditure is equivalent between the sexes [8,38]. For example, Caudwell and colleagues [8] reported body mass changes ranging from −14.7 to 2.0 kg in men and −10.0 to 4 kg in women. Furthermore, when participants are retrospectively classified as “responders” or “non-responders” (based on their actual weight loss relative to their predicted weight loss), there is some evidence supporting higher ad libitum energy intake in individuals experiencing lower than their predicted weight loss [29,37,38].

## 6. Implications and Future Directions

Scientific interest in potential sex-based differences in appetite regulation stems from initial evidence suggesting that men experience greater body mass and body fat reductions after exercise training than women [5,6]. Furthermore, evidence from an evolutionary biology perspective suggesting that women have evolved to store body fat to preserve energy balance and reproductive function has also driven research endeavour in this regard [10,12]. However, more recent experimental work has questioned the prevailing view that exercise is less effective for inducing weight loss in women, with several studies showing equivalent effects of exercise training on body composition in both sexes when the exercise-induced energy expenditure is matched [8,9]. Collectively, the balance of findings’ presented in this review suggest that men and women do not exhibit different responses (appetite, appetite-regulatory hormones, energy intake) to acute or chronic exercise-induced energy deficits. This has important implications for men and women engaging in exercise for health, and supports the promotion of exercise as a weight management tool for all. However, it is likely that women will need to exercise for a longer duration and/or at a higher intensity to achieve a similar exercise energy expenditure as men.

The findings provided within the current literature from experiments focussing on weight loss can be useful in informing individuals about exercise and dietary approaches for health. In this regard, it has been established that exercise-induced energy deficits stimulate smaller changes in appetite, appetite-regulatory hormones and energy intake compared with dietary restriction in both men [71,96] and women [15]. This may assist in informing an individuals’ decision regarding their preferred method of inducing an energy deficit for weight loss and also provide information regarding the anticipated homeostatic responses (i.e., greater appetite stimulation with food restriction).

Considering that exercise training appears to elicit at most a partial, but incomplete, compensation in energy intake in both sexes, it seems that men and women can endure prolonged periods in an exercise-induced energy deficit which may facilitate the development of a negative energy balance. Such incomplete compensation also supports evidence that larger exercise-induced energy deficits promote greater weight loss during an exercise intervention [34,35]. However, it is worth reiterating the considerable variability in responses (albeit to a similar degree in men and women), with some individuals appearing susceptible to increased hunger and energy intake with exercise training that attenuates the degree of weight loss [29,37,38]. Nevertheless, exercise training triggers marked improvements in other important outcomes in the absence of weight loss (e.g., cardiorespiratory fitness, body composition, insulin sensitivity) [26], which is important for those undertaking exercise for health benefits.

Although a considerable body of literature has developed understanding of the relationship between exercise, appetite and weight control, there are only a few studies which have directly focused on sex-based differences. Additional research is required to expand the evidence base before definitive conclusions can be drawn. This should include different types of exercise and insights into the mechanisms governing appetite control, both of which appear sparse in the current literature. Studies investigating a wider array of appetite parameters, particularly appetite hormones beyond the initiation of exercise training, between men and women would also be welcomed. Furthermore, considering potential sex-based differences in non-homeostatic factors governing energy balance (e.g., neuronal responses [100], and cognitive/behavioural cues [101]) is another important line of scientific inquiry and will provide a more holistic insight into appetite regulation in men and women. Future research into the appetite, appetite-regulatory hormone and energy intake responses of elite athletes to exercise and dietary interventions also represents an important future research direction to better understand energy balance and the consequences of energy manipulation in this population. It is imperative that acute and chronic investigations adopt mixed-measures designs and utilise objective measures of energy balance components when examining interactions among appetite, appetite-regulatory hormones and energy intake between men and women.

## 7. Conclusions

This review has demonstrated that appetite, appetite-regulatory hormone and energy intake responses to acute exercise-induced energy deficits are similar between men and women. Specifically, the consensus of evidence suggests that acute exercise transiently suppresses appetite, and does not stimulate compensatory changes in appetite, appetite-regulatory hormones or energy intake in the hours after acute exercise in either sex. Evidence derived from exercise training studies appear less conclusive, with limited evidence that women, but not men, respond to the initiation of exercise training with compensatory changes in appetite-regulatory hormones conducive to appetite stimulation. However, it is not known whether this change translates into long-term differences after a more sustained period of exercise training. Furthermore, evidence does not support a sex dimorphism in appetite or energy intake when assessed objectively, and increasing energy expenditure through exercise elicits at most a partial energy compensation in both sexes. Few studies have directly compared appetite, appetite-regulatory hormone and energy intake responses to acute and chronic exercise interventions between men and women. Therefore, these conclusions are supported by evidence drawn from the limited studies directly comparing the sexes and supplemented by those conducted in men and women separately. A better understanding of whether appetite, appetite-regulatory hormone and energy intake responses to exercise-induced energy deficits differ by sex may contribute to the development of more effective weight management strategies.
